# Review on the Pharmacological Properties of Phillyrin

**DOI:** 10.3390/molecules27123670

**Published:** 2022-06-07

**Authors:** Chenyu Zhou, Mengya Lu, Jialei Cheng, Emelda Rosseleena Rohani, Hamizah Shahirah Hamezah, Rongchun Han, Xiaohui Tong

**Affiliations:** 1 School of Pharmacy, Anhui University of Chinese Medicine, Hefei 230012, China; zhoucy@stu.ahtcm.edu.cn (C.Z.); 2020205225067@stu.ahtcm.edu.cn (M.L.); chengjialei@hotmail.com (J.C.); hanr@ahtcm.edu.cn (R.H.); 2 Institute of Systems Biology, Universiti Kebangsaan Malaysia, Bangi 43600, Malaysia; emelda@ukm.edu.my (E.R.R.); hamizahshahirah@ukm.edu.my (H.S.H.); 3 School of Life Sciences, Anhui University of Chinese Medicine, Hefei 230012, China

**Keywords:** phillyrin, pharmacological properties, toxicity, anti-inflammatory effects

## Abstract

Phillyrin is an effective lignan glycoside extracted from a traditional Chinese medicine *Forsythia suspensa* (Thunb.) Vahl (Oleaceae). It mainly exists in the roots, stems, leaves and fruits of the plant, with the highest content in the leaves. In terms of its medicinal application, there are a large number of experimental data proving its pharmacological effects in vitro and in animal models, such as anti-inflammatory, anti-obesity, anti-tumor, etc. Furthermore, pharmacokinetic experiments have also shown phillyrin’s high effectiveness and low toxicity. Despite more than one thousand studies in the literature on phillyrin retrievable from Web of Science, PubMed, and CNKI, few reviews on its pharmacological activities have been presented conclusively. In this paper, we aimed to summarize the pharmacological and pharmacokinetic characteristics of phillyrin from the current literature, focusing on its anti-inflammatory, anti-aging, antiviral, antibacterial, hepatoprotective and anti-cancer effects, hoping to come up with new insights for its application as well as future studies.

## 1. Introduction

*Forsythia suspensa* (Thunb.) Vahl (Oleaceae) is a traditional Chinese medicine first recorded in *Shennong Bencao Jing*, which was a book published ca. 2000 years ago documenting Chinese folk medicines. Forsythiae Fructus, the dried fruit of *F. suspensa*, is frequently used in China by physicians for heat clearing and detoxifying. Modern pharmacological studies showed that Forsythiae Fructus has antipyretic, anti-inflammatory, antiviral, antibacterial, as well as anti-tumor effects, and therefore, it is clinically used to treat fever, influenza, tumor, hypertension, and other diseases [1]. As the main active components of this medicinal plant, more than 50 lignans have been isolated and characterized from various organs of *F. suspensa* (fruit, flower, leaf and root) [2], with phillyrin (C_27_H_34_O_11_) as the key compound [3,4]. Phillyrin, a lignan glycoside, is the phytochemical marker for Forsythiae Fructus quality assessment in *Chinese Pharmacopoeia* 2020 edition, and it is stipulated that the content of phillyrin shall not be less than 0.15% when calculated as dry product [5].

Recently, phillyrin was proven to possess strong pharmacological effects against inflammation, oxidation, cancer, viral and bacterial infection, indicating its great potential in treating corresponding diseases. Regarding resources for phillyrin, it can be extracted from all parts of *F. suspensa*, although the purified contents and bioactivity of each part vary. A number of other medicinal plants, including *Osmanthus fragrans* [6], *O. heterophyllus* [7], *O. fragrans* var. *aurantiacus* [8] *Chionanthus virginicus* [9] and *C. retusus* [10], also contain phillyrin. Notably, the natural production of phillyrin is not confined to Oleaceae. *Lancea tibetica* [11], *Flammulina velutipes* [12], and even endophytic fungi inside *F. suspensa*, such as *Colletotrichum gloeosporioides*, also produce phillyrin and are precious natural resources for this pharmaceutical compound (Table 1) [13]. Despite its significant efficacy in treating diseases and the wide distribution in nature, phillyrin does not draw much attention from researchers worldwide.

As of 17 April 2022, using keywords phillyrin or forsythin (an alternative expression for phillyrin) to search the Pubmed database, we retrieved only 296 publications. Meanwhile, 1587 items were obtained with the same strategy in the China National Knowledge Infrastructure (CNKI) database. In this review, we combed the literature in the Web of Science, PubMed and CNKI public repositories with special interests in pharmacological and metabolic features of phillyrin. Articles published in and after 2012 were analyzed intensively for the better understanding of phillyrin and providing key information for future research.

## 2. Biology

**Figure 1:**
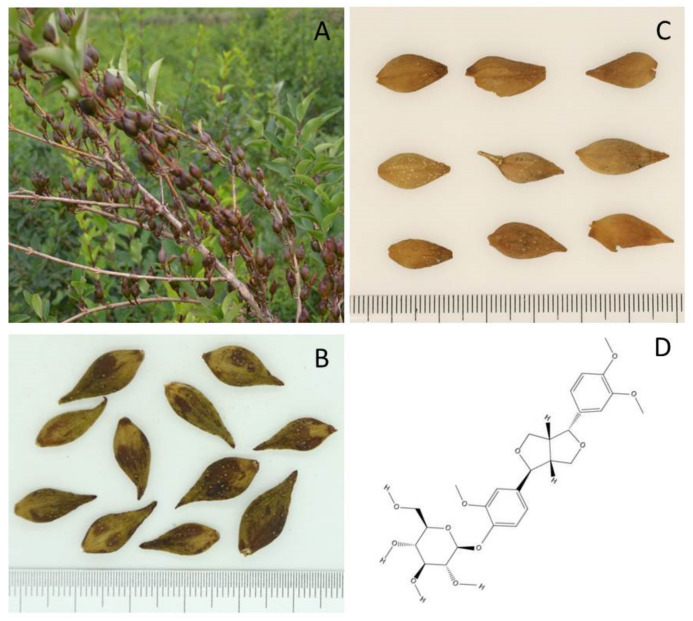
*F. suspensa* and phillyrin. (**A**) Fruits of *F. suspensa* grown in Lingchuan County, Shanxi Province, China; (**B**) Qing qiao; (**C**) Lao qiao; (**D**) Structure of phillyrin (created with ChemDraw 19.0).

*F. suspensa* is a deciduous shrub that is sometimes cultivated in gardens as an ornamental plant due to its beautiful yellow flowers blossomed in spring. With its branches pendulous or spreading, the plant has yellow–brown or gray–brown branchlets, and the internodes are hollow. The leaves are simple, and the shape of the leaf blade is ovate to broadly ovate. The flowers are solitary or two to several in leaf axils. With scattered lenticels, the capsules of the plant are ovoid to long ellipsoid (Figure 1A). Except for South China, *F. suspensa* is widely distributed in Eastern Asia [14].

As a crude drug recorded in *Chinese Pharmacopoeia*, the fruit of *F. suspensa* is usually categorized into two forms: unripe Forsythiae Fructus (Qing qiao) and ripe Forsythiae Fructus (Lao qiao) (Figure 1B,C). Using high-performance liquid chromatography (HPLC), Hu et al. found that the content of phillyrin in Qing qiao was significantly higher than that of Lao qiao from the same origin [15,16]. However, the content of phillyrin in the fruit is not as high as in other parts of the plant. Based on the comparison of reference substances and analysis of HPLC fingerprint for extracts from different parts of *F. suspensa* in the literature, Ma et al. demonstrated that Forsythoside A has maximum concentration in the fruits. Nevertheless, the content of phillyrin is much lower than that in the root and stem of *F. suspensa* [17]. When researchers turned their attention to the leaves, regarding the content of phillyrin, they reached the conclusion as follows: leaves (2.60%) > Qing qiao (0.91%) > Lao qiao (0.17%) [18]. To sum up, in addition to the traditional application of Forsythiae Fructus as medicine, the effective utilization of *F. suspense* leaves, roots and stems is of great significance as far as phillyrin extraction is concerned. Because of its physicochemical property, the extraction of phillyrin from various parts of *F. suspensa* often involves different concentrations of methanol or ethanol in combination with optimal solid–liquid ratio, extraction temperature and time to achieve high extraction efficiency. In addition, flash extraction and ultrasonic or microwave-assisted extractions are also frequently reported [19]. The yield of phillyrin with the above-mentioned approaches is generally satisfactory.

The biosynthesis of lignans in plant utilizes the phenylpropanoid pathway. Such compounds can be divided into the following types according to their molecular architecture: aryltetralin, dibenzylbutyrolactone, arylnaphthalene, furofuran, tetrahydrofuran and dibenzylbutane. Phillyrin is a type of furofuran lignan that features phenolic dimers containing a 2, 3-dibenzylbutane structure (Figure 1D). Phillygenin, an aglycon of phillyrin, can be obtained by enzymolysis with cellulase or hydrolysis.

## 3. Pharmacological Properties of Phillyrin

As one of the main bioactive components of *F. suspensa*, the pharmacological effects of phillyrin are significant and versatile. This review collected and sorted relevant literature on phillyrin experiments in China and abroad since 2012, which are presented in Table 2.

### 3.1. Effects on Metabolic Disorders

#### 3.1.1. Obesity

Obesity refers to an abnormal or excessive accumulation of fat, which is a well-known risk factor to diabetes, hypertension and other related metabolic diseases [45]. In vitro, phillyrin has shown an inhibiting effect on lipid accumulation induced by high glucose in the Hep G2 cell line [46]. It has also been proven in animal experiments that medium (53.2 mg/(kg m_b_·d)) and high (159.6 mg/(kg m_b_·d)) doses of phillyrin could significantly reduce the volume of adipocytes in adipose tissue in rats fed with high-fat diet (HFD) [22]. However, despite that there are many in vivo data supporting the anti-obese action of phillyrin, few studies in the literature have covered the underlying mechanisms. Xiao et al. speculated that phillyrin functioned as an AMPK activator, increasing the expression of PPARβ/δ and ANGPTL4 to promote fat metabolism and weight loss in HFD mice [20]. In addition, by using molecular docking technology, phillyrin was identified as an inhibitor of phosphodiesterase 3B (PDE3B). PDE is an enzyme that catalyzes the hydrolysis of cyclic adenosine monophosphate (cAMP). cAMP binds to the regulatory units of protein kinase A (PKA), allowing for lipolysis in adipocytes [47]. Thus, PDE3B might be a target for phillyrin-initiated weight loss in obesity [46]. Nevertheless, more efforts are needed for understanding the role of phillyrin better in the regulation of systemic metabolism.

#### 3.1.2. Diabetic Nephropathy (DN)

Previous reports suggested some potential for phillyrin in the treatment of DN, which is one of the common chronic complications of diabetes and the main cause of death in diabetic patients. Firstly, phillyrin has been shown to increase insulin sensitivity by promoting glucose uptake in insulin-resistant 3T3-L1 adipocyte via activation of the PI3K/Akt signaling pathway [21]. Secondly, phillyrin was also reported to significantly improve the pathological changes in kidneys in DN animals. After treatment with phillyrin, glomerular volume, basement membrane thickness and inflammatory cell infiltration were significantly reduced in diabetic rats, which was associated with a reduced expression of TGF-β_1_ in renal tissue of DN rats [41]. By activating the PI3K/Akt/GSK-3β signaling pathway, phillyrin inhibited glycogen synthase kinase-3β (GSK-3β) activity, thereby suppressing the activation of caspase-3 and ultimately preventing the apoptosis of renal cells [42]. Therefore, phillyrin might be applied as a therapeutic target of DN.

### 3.2. Anti-Inflammatory Effects

As the “holy medicine for sore family”, the anti-inflammatory effects of Forsythiae Fructus are consistent with the traditional usage of reducing swelling and dispersing knots. Extracted from the dried fruit of Forsythiae Fructus, phillyrin is composed of two phenylpropanoid side chains linked to each other. The oxygen bridge on the structure of phillyrin is one of the groups that plays the crucial role in exerting antioxidant, antibacterial and anti-inflammatory effects [26].

**Figure 2:**
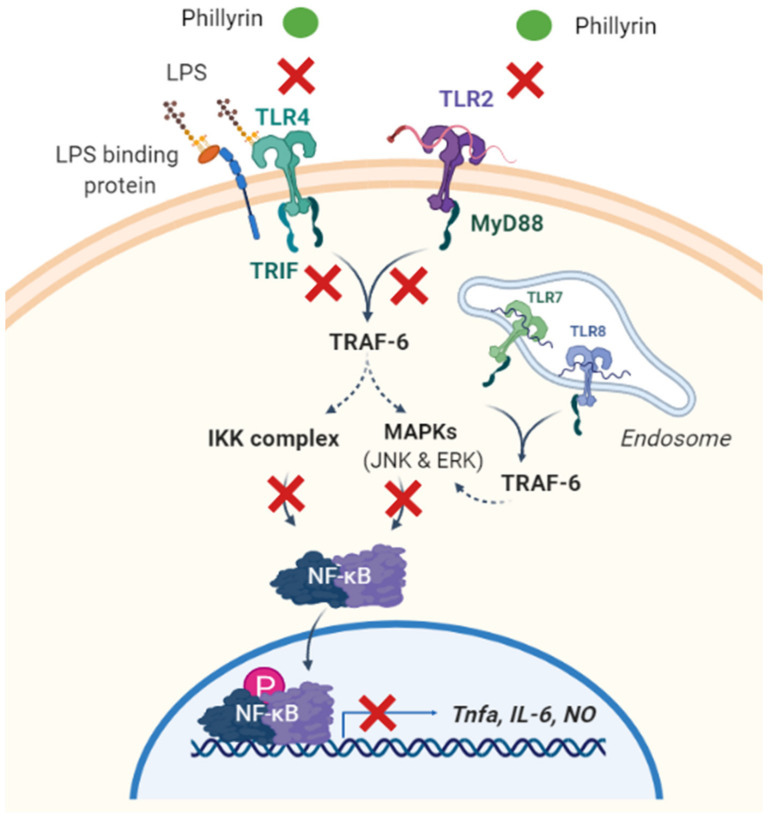
Graphic scheme of potential targets underlying phillyrin’s anti-inflammatory function. Phillyrin inhibits LPS-induced inflammation via the suppression of the TLR2 and TLR4 signaling pathways. Subsequently, in both MyD88-dependent and MyD88-independent manners, downstream NF-κB and MAPK signaling is inhibited by phillyrin, so as the production of pro-inflammatory cytokines. IKK: IKappaB kinase, IL-6: interleukin-6, LPS: lipopolysacharide, MyD88: myeloid differentiation primary response 88, NF-κB: the nuclear factor NF-kappaB, NO: nitric oxide, TLR: Toll-like receptor, TNF-α: tumor necrosis factor-α, TRAF6: TNF receptor-associated factor 6. (This figure was created with Biorender).

Inflammation is a kind of body defensive response to ensure that harmful stimuli are removed and damaged tissue is repaired. When immune cells are under the action of inflammatory factors, certain soluble proteins or peptides with small molecular weight, which can transmit information between cells and have specific immune regulation function, are secreted by the body and can participate in or cause inflammatory reactions. These substances are called inflammatory factors, such as NO, TNF-α, IL-6, PGE2, IL-1, and so on [48]. Phillyrin changes the expression level of these inflammatory factors by influencing the inflammation-related signaling pathways (Figure 2).

#### 3.2.1. NF-κB Signaling Pathway

NF-κB is a eukaryotic cell transcription factor, belonging to the Rel family, and it widely exists in various mammalian cells. It is full-featured to be involved in the inflammatory response and stress response, cell cycle regulation, cell apoptosis, growth and proliferation [49], and lymphocyte development and communication between cells [50]. Traumatic disease in many organs can lead to an inflammatory response. In the central nervous system, microglias are most densely distributed in the substantia nigra. When brain tissue is stimulated, microglias are activated and release inflammatory factors and free radicals upon arrival at the site of injury. These factors can not only directly induce neuronal apoptosis and cerebral edema but also lead to the destruction of the blood–brain barrier. Parkinson’s disease (PD) and traumatic brain injury (TBI) are the results of microglial activation. In a mouse model of TBI, phillyrin activated the PPARγ signaling pathway and showed anti-inflammatory effects by preventing NF-κB phosphorylation and inhibiting the release of its downstream pro-inflammatory factors in microglias [23]. In the study of acute kidney injury (AKI), as an upstream signal of inflammatory pathways, NF-κB and MAPK were activated by a series of inflammatory responses associated with AKI, leading to the production of large amounts of downstream inflammatory cytokines such as IL-6, TNF-α and IL-1β [24]. The NF-κB pathway activated by lipopolysaccharide (LPS) can activate hepatic stellate cells (HSCs) and enhance their proliferation and migration. In vitro experiments showed that phillyrin inhibited the LPS-induced phosphorylation of NF-κB P65 protein in HSC-T6 cells, thereby preventing HSCs activation [30]. Given these findings, it is still necessary to further identify the proteins that interact with phillyrin through quantitative proteomics and other techniques in order to find new drug targets that can regulate inflammation.

#### 3.2.2. Toll-Like Receptors 4 (TLR4)

TLR4 is currently considered to be the initiating gate of inflammatory response and a key molecule that causes inflammation in the body. After binding with TLR4 on the target cell membrane, the transmembrane signal activates transcription factors such as NF-κB through the intracellular domain of TLRs, and it further induces the release of multiple inflammatory molecules [31]. LPS is one of the main pathogenic factors of Gram-negative bacteria, which can cause a variety of acute and chronic inflammatory reactions in humans and animals, resulting in tissue and organ damage [27].

In an LPS-induced macrophage RAW264.7 inflammation model, the mechanism is considered to be that LPS activates its primary receptor TLR4, which triggers the innate immune system to initiate a cascade of signals, leading to the activation of MAPK, which induces the secretion of pro-inflammatory cytokines such as NO, TNF-α and IL-6. The research groups used molecular docking technology to prove that the active site of phillyrin and TLR4 was stably bound, with good affinity between the two, and then indicated that the protective effect of phillyrin on acute inflammation-induced lung injury may be through the influence of the TLR4 signaling pathway from the result of animal and cell experiments [26]. Similarly, LPS-stimulated microglia BV-2 released inflammatory factors and free radicals such as superoxide anion, NO, prostaglandin and other neurotoxic factors after arriving at the site of injury, enhancing oxidative stress and causing progressive degeneration and necrosis of neurons. The protective mechanism after treatment of phillyrin was mainly ascribed to the downregulation of the LPS signal transduction pathway key receptor TLR4 expression [27]. There are more than 20 kinds of TLRs distributed in cells, among which TLR2 and TLR4 are only expressed in myeloid cells, such as mononuclear macrophages [51]. It has been reported that *Staphylococcus aureus* can affect the function of human mononuclear macrophages by regulating the expression of TLR2 and TLR4. Phillyrin significantly decreased the secretion of TNF-α, IL-6, IL-8 and McSf-1 induced by *S. aureus*, and it inhibited the expression of TLR2 and TLR4, but the downstream signaling molecules involved need to be further explored [31].

NF-κB is a key transcription factor downstream of the TLR4 signaling pathway. The TLR signaling pathway can be divided into MyD88-dependent and MyD88-independent according to whether MyD88 mediates, while TLR4 has functions related to the above two signaling pathways [52]_._ In the LPS-treated mouse mammary epithelial cells (MECs), researchers demonstrated that phillyrin inhibits TNF-α and IL-6 secretion by inhibiting the TLR4/MyD88/TRaf-6/NIK pathway or activating the TLR4/MyD88/Traf-6/IκB pathway [29].

#### 3.2.3. MAPK Signaling Pathway

The MAPK signaling pathway is a pivotal signal transduction system that mediates cell proliferation, stress, inflammation, differentiation, functional synchronization, transformation and apoptosis. There are at least three subsets of MAPK, including ERK c-Jun N-terminal Kinase (JNK) and p38 kinase families [53]. Among them, the activation of ERK is mainly closely related to the initiation of growth factors and is involved in cell proliferation and differentiation. JNK is a key molecule involved in various stress responses in the body, and P38 principally mediates inflammation and apoptosis-related pathways [54]. Phillyrin at 40 µM significantly inhibited the phosphorylation and activation of ERK1/2 and JNK in 3T3-L1 adipocytes treated with TNF-α [55]. Wang et al. also showed that phillyrin significantly inhibited RANKL-induced osteoclastogenesis of BMM cells in vitro and suppressed LPS-induced calvarial osteolysis in mice. Osteoclast formation is closely linked to the NF-κB and MAPK pathways. Phillyrin dose-dependently attenuated the activation and phosphorylation of ERK and JNK to prevent LPS-induced osteolysis in mice while having no effect on p38 [56].

### 3.3. Anti-Aging Effect

According to the free radical aging theory, the human body produces free radicals in the process of life activities, and the chain reaction of free radicals leads to the damage of the biofilm and the cross-linking of biological macromolecules. As a result, protein and nucleic acid molecules are damaged, so that biofilms fail to function and lipofuscin accumulates, leading to aging and death [57]. In a mouse model of aging induced by D-galactose, phillyrin significantly increased the activities of SOD, T-AOC and GSH-Px in the serum, liver and brain of aged mice, reducing the accumulation of MDA and the activity of MAO-B, and protecting the body from free radical damage [34].

### 3.4. Antiviral Effects

In modern pharmacology, the antiviral effects of phillyrin are basically consistent with the heat-clearing and detoxifying effect of Forsythiae Fructus recorded in ancient prescriptions. Phillyrin can inhibit the nuclear protein (NP) gene expression of influenza A virus after transfection so as to achieve the anti-“A flu” effect [1]. Other studies have shown that phillyrin can significantly prevent the replication of influenza A virus [35]. It is not only influenza A virus but also the devastating coronavirus (COVID-19), the cause of the global public health crisis in the past two years, that has brought phillyrin as an effective remedy into the field of scientific research again [58]. Based on a bioinformatics analysis, researchers identified 192 common core targets and 25 biological pathways for phillyrin in the treatment of SARS-CoV-2 and influenza virus co-infection. It is concluded that HIF-1, PI3K-AKT and RAS may be the main signaling pathway of the antiviral effect of phillyrin, which provides a new idea for follow-up COVID-19 treatment.

### 3.5. Antibacterial Effects

Forsythiae Fructus holds certain inhibitory effects on bacteria and has a wide antibacterial spectrum. As one of its main active components, phillyrin can antagonize bacterial endotoxin [31]. In vitro experiments showed that phillyrin had the potential to inhibit the production of quorum sensing regulatory virulence factors such as pyocyanin, elastase, and rhamnolipid in *P. aeruginosa*. The results suggest that phillyrin can be used as an alternative of antibiotics against bacterial infection [36]. Phillyrin also shows therapeutic effects on *K. pneumonia*-induced diarrhea, pneumonia, and urinary tract infections. The addition of phillyrin can activate the STAT5/Foxp3 pathway in *Kp*-infected mice with pneumonia to promote Treg differentiation and to reduce the level of inflammatory factors. The imbalance of Th17/Treg cell ratio was alleviated for protecting *Kp*-infected pneumonia mice [37]. In the rat perianal abscess model, phillyrin could significantly reduce the number of *Escherichia coli* colonies on the wound surface, and the researchers proposed that the mechanism of action of phillyrin might be related to activation of the Janus kinase/Signal transducer and activator of transcription 3 (JAK2/STAT3) signaling pathway [38].

### 3.6. Hepatoprotective Effects

Chronic hepatitis is a long-term chronic liver injury and inflammation caused by a variety of pathogenic factors, and fibrosis is the most important pathological change in the development of chronic hepatitis. According to the results of animal experiments, phillyrin can ameliorate CCL4/ANIT-induced inflammation, fibrosis and injury in experimental animals. The mechanism may be related to the inhibition of the NF-κB signaling pathway and TGFβ_1_/SMad_2/3_ signaling pathway, which was further verified in vitro in hepatic stellate cells [39]. In the liver of patients with alcoholic hepatitis, alcohol metabolite acetaldehyde can directly cause the apoptosis of hepatocytes and thus increase reactive oxidative species (ROS) levels. Studies have shown that phillyrin exhibited a hepatoprotective effect by reversing alcohol-induced apoptosis in the liver in vivo [40]. However, the molecular mechanisms of this protective effect remain elucidated.

### 3.7. Anti-Cancer Effects

Researchers also suggested that phillyrin has striking anti-tumor potential based on the links between tumor pathogenesis and proven pharmacological effects of phillyrin. For example, according to an experiment studying laryngeal squamous cell carcinoma (LSCC), phillyrin alone has almost no effect on the proliferation and apoptosis of HEp-2 cells, but it can significantly improve the autophagy level of HEp-2 cells. A growing number of studies have shown that various inflammatory factors lead to the occurrence and metastasis of tumors by participating in and changing the formation of the microenvironment [59]. Combined with the anti-inflammatory effect of phillyrin, this induction of autophagy in tumor cells may be related to the AMPK/mTOR/p70S6K pathway [44]. From another study of Lewis lung carcinoma, in vivo experiments in mice showed that phillyrin inhibited VEGF specifically expressed in lung cancer tissue, thereby inhibiting further tumor angiogenesis. In addition, hematoxylin and eosin (HE) section staining showed that a high dose of phillyrin could significantly decrease the volume and tissue density of lung tumors. It is suggested that phillyrin may be a potential active ingredient for inhibiting the development of lung cancer, although the specific mechanism needs further research [43].

## 4. Metabolism and Tissue Distribution

**Figure 3:**
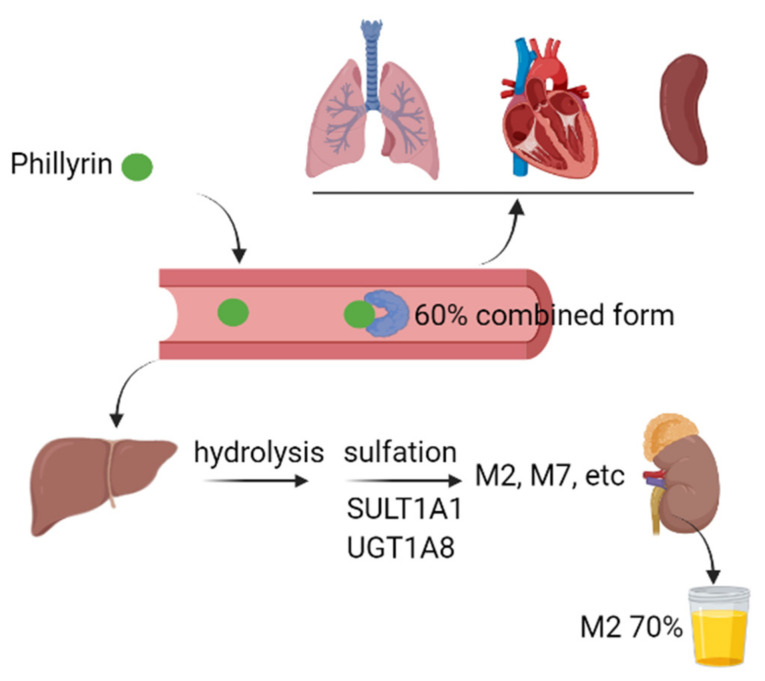
Graphic scheme of pharmacokinetics of phillyrin in vivo. The plasma protein binding rate of phillyrin reached around 60% upon absorption. After absorption, phillyrin was widely distributed in heart, lung, spleen, liver and kidney. In liver, phillyrin underwent extensive hydrolysis and sulfation catalyzed by enzymes such as SULT1A1 and UGT1A8. Most of the parent component and its metabolites were mainly secreted via urine. SULT1A1: sulfotransferase 1A1, UGT1A8: UDP-glucuronosyltransferase 1A8. (This figure was created with Biorender.)

We summarized the pharmacokinetic properties of phillyrin in experimental animals and human (Figure 3). By using ultrafiltration and HPLC, the plasma protein binding rate of phillyrin was found to be approximately 60%, regardless of its total concentration in plasma in SD rats [60]. The other study equipped with UPLC-Q-TOF-MS demonstrated that phillyrin undergoes hydrolysis, oxidation and sulfation in the liver, and its metabolites from these biotransformation pathways are secreted from bile, urine and feces in rats [61]. Pan et al. further demonstrated that there were similarities in the metabolic pathways of phillyrin between humans and experimental animals. Firstly, hydrolysis was the initial and main metabolic pathway of phillyrin, which was followed by extensive sulfation to form M2 and a reduced level of glucuronidation to form M7. Secondly, the plasma exposure of M2 and M7 are 86- and 4.2-fold higher than that of phillyrin. Within 48 h, approximately 75% of the administrated dose is found in urine, with M2 accounting for around 70%. In addition, the authors also revealed that sulfotransferase 1A1 and UDP-glucuronosyltransferase 1A8 are the most active hepatic enzymes involved in the formation of M2 and M7, respectively [62]. In the research on drug pairs of Flos Lonicerae and Forsythiae Fructus, the content of phillyrin in different organs was determined by HPLC after oral administration of specified dosages. Although phillyrin was detected in the heart, liver, spleen and lung, its concentration reached the highest in the kidney 2 h later [63]. Thus, further investigations are needed for better understanding of the pharmacokinetic properties of phillyrin in vivo.

## 5. Toxicology

Studies on the toxicity of phillyrin have rarely been reported, and only in recent years has it attracted the attention of the medical community. In animal experiments, the researchers conducted toxicity tests of phillyrin on non-rodent Beagle dogs, and they found that some animals in the high-dose group had soft or loose stools during the administration period. Meanwhile, some dogs had vomiting and leftover food after the administration, which returned to normal during the recovery period [64]. Similarly, in the acute toxicity test of mice, a small number of animals showed symptoms such as immobility, vertical hair and eye closure, which all returned to normal within 30 to 60 min after administration, and no death occurred [65]. Therefore, it is inferred that the gastrointestinal reactions may be caused by long-term administration of large doses of test materials. Results of subchronic toxicity tests in rats for up to 30 days also showed that no deaths or significant toxicological effects were observed [66]. In addition, the teratogenic and mutagenic effects of phillyrin on animals are rarely reported. In conclusion, phillyrin oral administration has low or no toxicity and has great potential for the further development of food and drug applications.

## 6. Conclusions

The annual consumption of Forsythiae Fructus in China is more than 6000 metric tons, and in comparison to this, reports on phillyrin, its main active component was less than 300 in PubMed. The resource for phillyrin is ample and its efficacy is evident. Meanwhile, the toxicity of phillyrin is low. More research needs to be conducted for this promising yet underestimated compound. As the featuring compound extracted from Forsythiae Fructus, the capability of phillyrin coincides with its traditional usage, which is mainly “clearing away heat and toxic materials”. Among the many therapeutic effects, the ability of phillyrin to treat inflammation is the keyword. By searching literature reports, studies on the pharmacological activity of phillyrin in recent years mainly focus on inflammation-related diseases. With the outbreak of COVID-19, we also need to focus on the antiviral benefits of phillyrin. Moreover, growing evidence indicate a potential for this component to be used in the treatment of metabolic disorders, such as obesity, diabetes, etc. Another observation is that phillyrin and the extract mixture of Forsythiae Fructus containing more than 230 chemicals share key pharmacological features including anti-inflammatory, antibacterial, antiviral and anti-cancer effects [19]. The scientific community will benefit in the future from profound investigations on the relationship between phillyrin and the fruit extract of *F. suspensa*. Taken together, extensive mechanical studies are required for the thorough understanding and pharmacological development of this compound.

## Figures and Tables

**Table 1:** Natural sources of phillyrin.

Species	Region	Reference
Oleaceae		
*Forsythia suspensa* (Thunb.) Vahl	Japan, Korea, China	[1]
*Osmanthus fragrans* (Thunb.) Lour.	China	[6]
*Osmanthus heterophyllus* (G. Don) P. S. Green	Japan, China	[7]
*Osmanthus fragrans var. aurantiacus* Makino	China	[8]
*Chionanthus virginicus* L.	Japan, Korea	[9]
*Chionanthus retusus* Lindl. et Paxt.	Japan, Korea, China	[10]
Other sources		
*Lancea tibetica* Hook. f. et Thoms	India, China	[11]
*Flammulina velutipes* (Curt. ex Fr.) Sing	Cultivated globally	[12]
*Colletotrichum gloeosporioides* (Penz.) Penz. et Sacc.	Distributed worldwide	[13]

**Table 2:** Pharmacological properties of phillyrin and possible mechanisms.

Models	Mechanism	Reference
Effects on metabolic disorders		
Mice fed with HFD	Phillyrin lowered body weight via the modulation of PPARβ/δ–ANGPTL 4 signaling pathway.	[20]
3T3-L1 adipocytes	Phillyrin promoted glucose uptake in insulin resistance 3T3-L1 adipocyte through activation of PI3K/Akt signaling pathway.	[21]
Rats fed with HFD	Not available	[22]
Anti-inflammatory effects		
Mouse model of traumatic brain injury	Phillyrin activated PPARγ signaling pathway to inhibit phosphorylation of NF-κB and its downstream pro-inflammatory action in microglia.	[23]
Mouse model of acute kidney injury induced by LPS	Phillyrin inhibited the activation of the NF-κB and MAPK signaling pathway, decreasing the levels of inflammatory cytokines (TNF-α, IL-1β, IL-6).	[24]
Lethal LPS-induced neutrophil Inflammation in zebrafish	Phillyrin reduced neutrophil infiltration, necrosis and inflammation via suppression of MyD88–NF-κB signaling pathway.	[25]
LPS-treated RAW264.7 cells; LPS-induced acute lung injury	Phillyrin inhibited the secretion of IL-6 and NO in RAW264.7 cells via TLR4 signaling pathway.	[26]
LPS-treated BV2 microglia cells	Phillyrin downregulated the expression of TLR4.	[27]
Periodontitis rats	Phillyrin reduced the phosphorylation of p38 MAPK and the expression of c-Fos.	[28]
LPS-treated mouse mammary epithelial cells	Phillyrin may decrease the secretion of inflammatory cytokines via inhibition of TLR4/MyD88/Traf-6/NIK or the TLR4/MyD88/Traf-6/IκB pathway.	[29]
LPS-treated rat hepatic stellate cells	Phillyrin restrained the expression of phosphorylated NF-κB p65 protein to inhibit HSC-T6 activation.	[30]
THP-1 cells stimulated with Staphylococcus aureus in vitro	Phillyrin inhibited the expression of TLR2 and TRL4.	[31]
Atherosclerosis in SD rats	Phillyrin reduced oxidative stress via decreasing gene and protein expression of NHE-1.	[32]
Traumatic fracture in SD rats	Phillyrin reduced the serum levels of inflammatory factors such as iNOS, TNF-α and IL-6.	[33]
Anti-aging effect		
Mouse model of aging induced by D-galactose	Phillyrin enhanced the activity of SOD and decreased the activity of MAO-B to improve the ability of scavenging free radicals in mice to inhibit aging.	[34]
Antiviral effect		
Mice infected with influenza A virus	Phillyrin may reduce inflammation induced by influenza A virus and inhibit viral replication.	[35]
Antibacterial effect		
Caenorhabditis elegans–Pseudomonas aeruginosa infection model	Phillyrin possibly suppress pathogen virulence factors to protect Caenorhabditis elegans from Pseudomonas aeruginosa.	[36]
Klebsiella pneumonia (Kp) infected mice	Phillyrin activated STAT5/Foxp3 pathway in Kp infected mice to promote the balance of Th17/Treg cells and relieving the disease.	[37]
Wound surface of rats with perianal abscess	Phillyrin reduced the number of Escherichia coli in the wound of perianal abscess rats by activating JAK2/STAT3 pathway.	[38]
Hepatoprotective effects		
Mouse model of liver fibrosis	Phillyrin inhibited NF-κB and TGF-β_1_/Smad_2/3_ signal pathway to repress the inflammatory response of macrophages and the activation of hepatic stellate.	[39]
Human liver cell line LO2 treated with alcohol	Phillyrin inhibited the expression of apoptosis related proteins PARP and Caspase 3.	[40]
Nephroprotective effects		
Diabetic nephropathy in rats induced by HFD and streptozotocin	Phillyrin inhibited inflammation and alleviated renal injury associated with depressed TGF-β1 expression.	[41]
Diabetic nephropathy in mice induced by streptozotocin	Phillyrin suppressed renal cell apoptosis via activation of PI3K/Akt/GSK-3β signaling pathway in kidney.	[42]
Anti-cancer effects		
Lewis lung carcinoma mice	Phillyrin inhibited lung tumor development by downregulating VEGF expression and upregulating endostatin expression, respectively.	[43]
HEp-2 cells	Phillyrin-induced autophagy may be through the AMPK/mTOR/p70S6K signaling pathway.	[44]

## Data Availability

Not applicable.
